# Excellence in Organ Utilisation—A Quantitative and Qualitative Evidence Base for a New Approach in the UK

**DOI:** 10.3389/ti.2023.11641

**Published:** 2023-09-04

**Authors:** Claire Williment, Jessica Jones, John Forsythe, Lisa Mumford, Stephen Powis

**Affiliations:** ^1^ NHS Blood and Transplant, Filton, United Kingdom; ^2^ NHS England, London, United Kingdom

**Keywords:** organ utilisation, equity, patient and clinical engagement, transplant strategy and policy, NHS transplant service

## Abstract

The Department of Health and Social Care in England established an Organ Utilisation Group, to collate and analyse evidence regarding the organ transplantation care pathway, make recommendations on how to reduce inequity of access, make the best use of available resources, and drive innovation in organ transplantation. The group consulted with national and international experts and stakeholders, sought views from service providers across the transplant care pathway, and heard from over 600 people, including over 250 patients, carers, and donors. The group uncovered new evidence about where improvements are needed—particularly in relation to patient experience and inequities in access. The final report suggests a new direction for organ transplantation services in the United Kingdom, with action required at local, regional, and national levels. Ultimately, it is expected to increase transplant activity through increased organ utilisation and improve patient experience, outcomes, and empowerment whilst also supporting the transplant clinical community.

## Graphical Abstract

**Figure:**
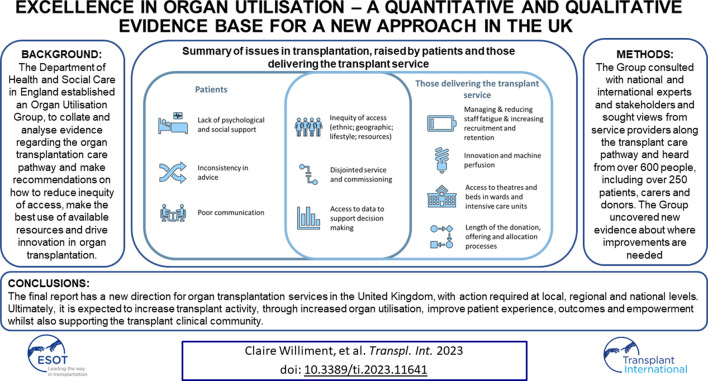

## Introduction

Over the last decade, around the world, most public facing programmes for transplantation have focused on the act of deceased donation or, where cultural mores make this challenging, living donation to allow a lifesaving or life enhancing transplant to occur [1].

There have been some remarkable successes in these programmes and there are some similarities in the way in which infrastructure for donation is augmented, often built on lessons learned from the Spanish system. These bring about improvement in donor numbers. But there are also national differences—not least in the ratio of deceased to living donation, organ specific “transplant per million population” achievements and the ratio of donation after death confirmation by neurological means (often termed Donation after Brain Death—DBD) compared with donation after death confirmation by circulatory cessation (often termed Donation after Circulatory Death—DCD) [1].

The last few years have also seen the trend of the increasing age of donors and increasing obesity in affluent countries [2]. The latter can bring problems in particular forms of donation such as liver and heart. Also, reflecting age and disease characteristics of the whole population, there is increased comorbidity in donors [3]. This has required transplant clinicians to investigate the safety and utility of using organs from patients with infection, tumour, and disease affecting other areas of the body [4–6]. There is an important principle of consent from transplant recipients who are being asked to accept an organ with a different risk profile compared with those that were transplanted a few years ago.

In May 2020, in the midst of the pandemic, England moved to a “deemed consent” basis for organ donation. The change in legislation [7]—which had strong public and clinical support—together with a wish to honour the choice of donors and their families, has led to a close examination of organ utilisation across the United Kingdom.

It was noted that, as in many countries, comprehensive planning arrangements have been put in place for organ allocation algorithms. Yet, a particular offer for a named patient is often made in the small hours of the morning, the final decision to accept or not frequently rests with a single clinician, and there is variable input from the patient themselves. The risk appetite of a particular clinician will naturally vary from time to time and based on the recent experience of that clinician in transplantation.

Preliminary examination of the UK wide data demonstrated differing acceptance rates from centre to centre and in access to innovative techniques that enhance utilisation.

## Materials and Methods

The Department of Health and Social Care (DHSC) established an Organ Utilisation Group (OUG), comprised of a range of subject matter experts and Chaired by NHS England’s Medical Director, to make recommendations on how to maximise the potential for organ transplantation from living and deceased donors, through making the best use of available resources, driving improvements to the infrastructure, and supporting innovation. Each person (whether a leader, member, or participant in a meeting or event) either added this project to their normal daily tasks or gave their time freely. All meetings were held online, which also enabled broader participation and accessibility.

**FIGURE 1:**
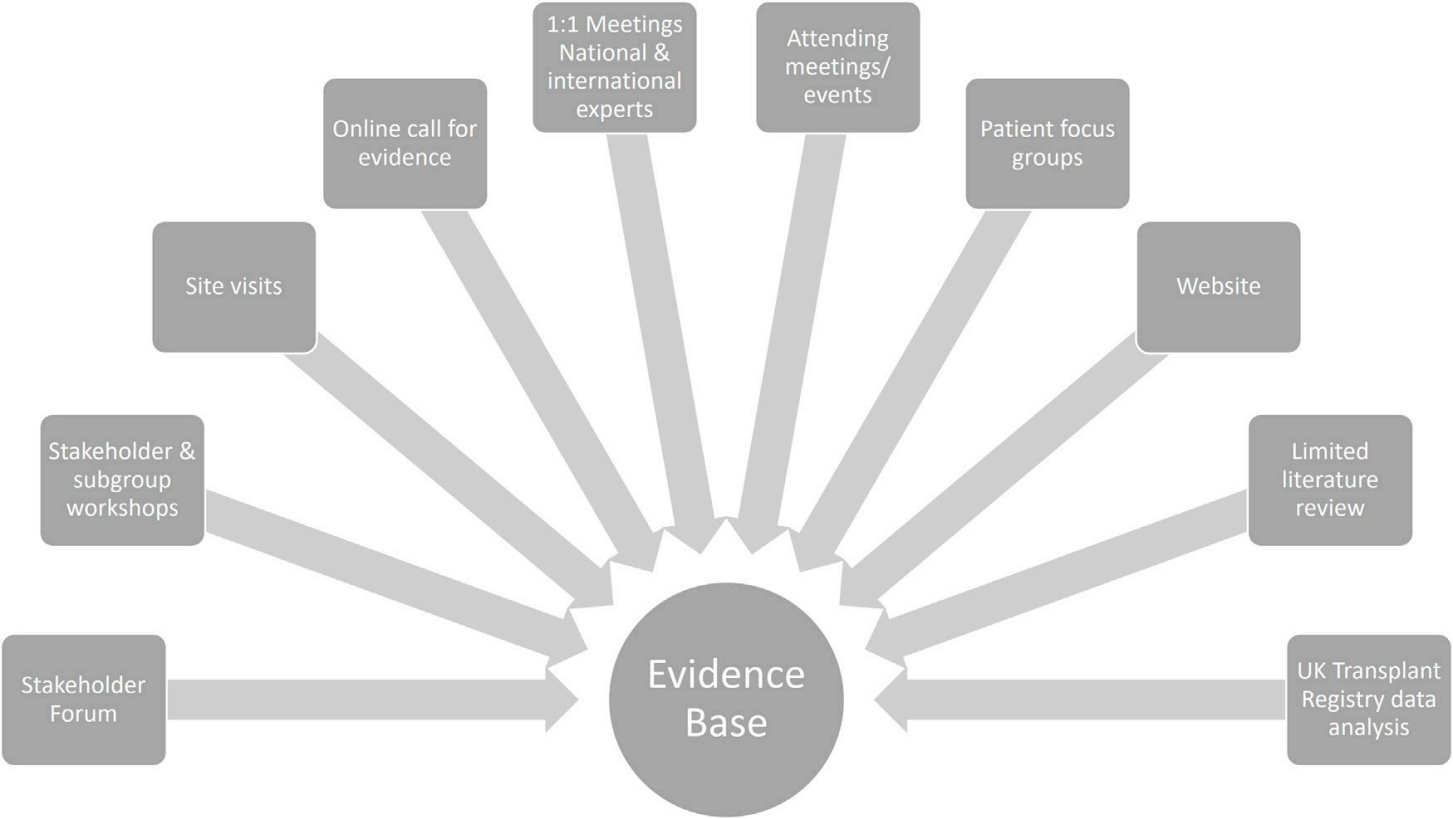
Organ utilisation group engagement and evidence gathering activities.

The OUG undertook work to identify the barriers to transplantation, examining national and international practice. This included patient focus groups, site visits, meetings with expert advisors, and reviews of the available data and literature. Figure 1 summarises the activities undertaken.

The OUG received responses from national and international transplant service providers, patients, carers, commissioners, professional organisations, charities, and patient representative groups through a range of routes:

• 97 responses to online call for evidence
• 248 responses to online patient survey
• 4 patient focus groups held with a total of 27 delegates
• 58 delegates at stakeholder workshop
• Meetings with international colleagues from 6 countries
• 22 members of the Stakeholder Forum
• Senior transplant leaders from 7 countries
• 10 site visits with representatives including senior management, clinical leaders, transplant surgeons, intensive care, recipient co-ordinators, physicians, psychologists, social care workers
• Wide range of stakeholder meetings, including: psychologists; social care workers; histopathology; Histocompatibility and Immunogenetics; transplant teams; clinical advisory groups; digital data provision experts; commissioners; Government transplant advisory groups; NHS Blood and Transplant organ-specific patient advisory groups; patient representative groups (including patient charities and support groups); community leaders (e.g., faith/belief leaders, community-specific champions).

The online call for evidence was open to the public and stakeholder groups and the OUG invited charities, patient, and clinical representative organisations to share with their members. It is therefore not possible to know the final number of people who received the survey and responded.

There was a remarkable consistency of views among patients, transplant teams and managers, backed by the data analysis, about the problems with transplantation and the opportunities to deliver improvements.

Data were extracted from the UK Transplant Registry held by NHS Blood and Transplant (NHSBT). This includes data on all patients waiting for or in receipt of a solid organ transplant in the United Kingdom. The number of organs donated per deceased donor were calculated, between 1 October 2020 and 31 March 2023. In order to evaluate the unwarranted variation across centres, offer decline rates were calculated by centres using offers from DBD donors, between 1 April 2019 and 31 March 2022, who had at least one heart retrieved, offered directly, and resulting in a transplant. Adult risk-adjusted median waiting times by centre were calculated for patients listed for a kidney between 1 April 2016 and 31 March 2019 using a Cox-proportional hazards model. Risk-adjusted death censored graft survival following deceased donor pancreas transplant between 1 April 2013 and 31 March 2017 was estimated using a Cox-proportional hazards model. Risk-adjusted 5 years patient survival from listing for adult elective liver registrations between 1 January 2010 and 31 December 2021 were estimated using a Cox-proportional hazard model.

## Results

### Feedback From an Online Call for Evidence

The OUG issued an open, online, call for evidence. The transplant community welcomed the opportunity to engage and the following responses were received:

• 74 Separate Respondents providing 93 responses in total.
• 107 challenges (+7 not applicable to OUG remit).
• 73 opportunities (+4 not applicable to OUG remit).
• 5 additional responses submitted via means other than the survey

Respondents were well dispersed across the UK. A chart of the residency of the individual giving a response is noted in Supplementary Figure S1 of the Supplementary Material.

Respondents were asked to categorise their comments as either a “challenge” or “opportunity” to improve the service. Frequently, respondents gave much more detailed information over and above a simple categorisation of an issue. The overall categorisation is summarised in Tables 1, 2.

**TABLE 1:** Summary of challenges in organ utilisation raised through the online call for evidence.

Challenge	N
Workforce (Staffing; fatigue; recruitment; sustainability)	20
Access to theatre and/or intensive care units	20
Data access (digital; imaging)	8
Risk aversion	7
Length of donation process	5
Lack of psychological support	5
Commissioning structure	4
Access to waiting lists	4
Offering process	3
Allocation process	3
Machine perfusion	3
Lack of patient education	3
Use of extended criteria organs	2
Workforce for research	2
Retrieval	2
Living donor liver transplantation	2
Pathology	2
Scouting	1
Inequity of access	1
Multi-Disciplinary Teams	1
Islet	1
Donation process	1

**TABLE 2:** Summary of opportunities for improving organ utilisation raised through the online call for evidence.

Opportunity	N
Machine perfusion/novel technology	26
Data provision	10
Standards/guidance	7
Buddying scheme	6
Commissioning structure	6
Structured decline review scheme	5
Pathology (PITHIA)	4
Patient choice/education	4
Scouts	3
Bring donation & Transplant communities closer	3
Team restructure	3
Psychological support	3
workforce/job description	3
Shared decision making	2
Strategic direction/leadership	2
Theatre/ITU access	2
Ethics Committee	1
Allocation	1
Paediatric liver	1

Particular focus was given to three aspects of the service:

1. Commissioning of referral for transplantation and the transplant procedure itself, especially the UK system of commissioning renal transplantation.
2. Standardisation of meetings that examined the decline of organs and peer review of units. The data available to units regarding the outcome of declined organs was mentioned frequently.
3. Damage of organs during retrieval—both avoidance of such injury to improve utilisation and better resolution of different views between retrieval and transplant surgeons.

### Feedback From Patients and Family Members/Carers

An online survey was issued in February 2022, to seek views from people who were waiting for, or had, transplants, and their families/carers. A key aim was to capture views from “less heard voices”—particularly Asian and Black female patients. Respondents were asked to rate different aspects of their care using a “star” rating, where 1 star was poor quality of care and 5 stars the highest quality of care. The survey was anonymous and covered both deceased and living donation. Respondents were given the option to record their ethnicity, but this was not a required field for completion.

**FIGURE 2:**
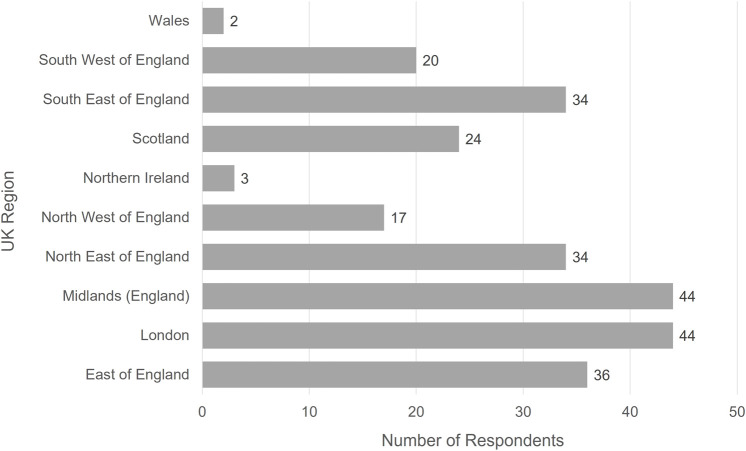
Geographical region of where respondents to the online patient survey received the majority of their care.

There were 258 responses received from people from across the UK (see Figure 2). Of the respondents:

• 193 had received a transplant.
• 26 were on the waiting list.
• 42 were family members/carers of those either on the waiting list or have received a transplant.
• 252 were answering as or on behalf of an adult, with 6 people answering on behalf of a child.
• 19 respondents had received a kidney/liver transplant from a living donor. Of these respondents, 14 people received their organ from a family member or friend, and 1 person received their organ from someone who responded to a media/social media appeal.

**FIGURE 3:**
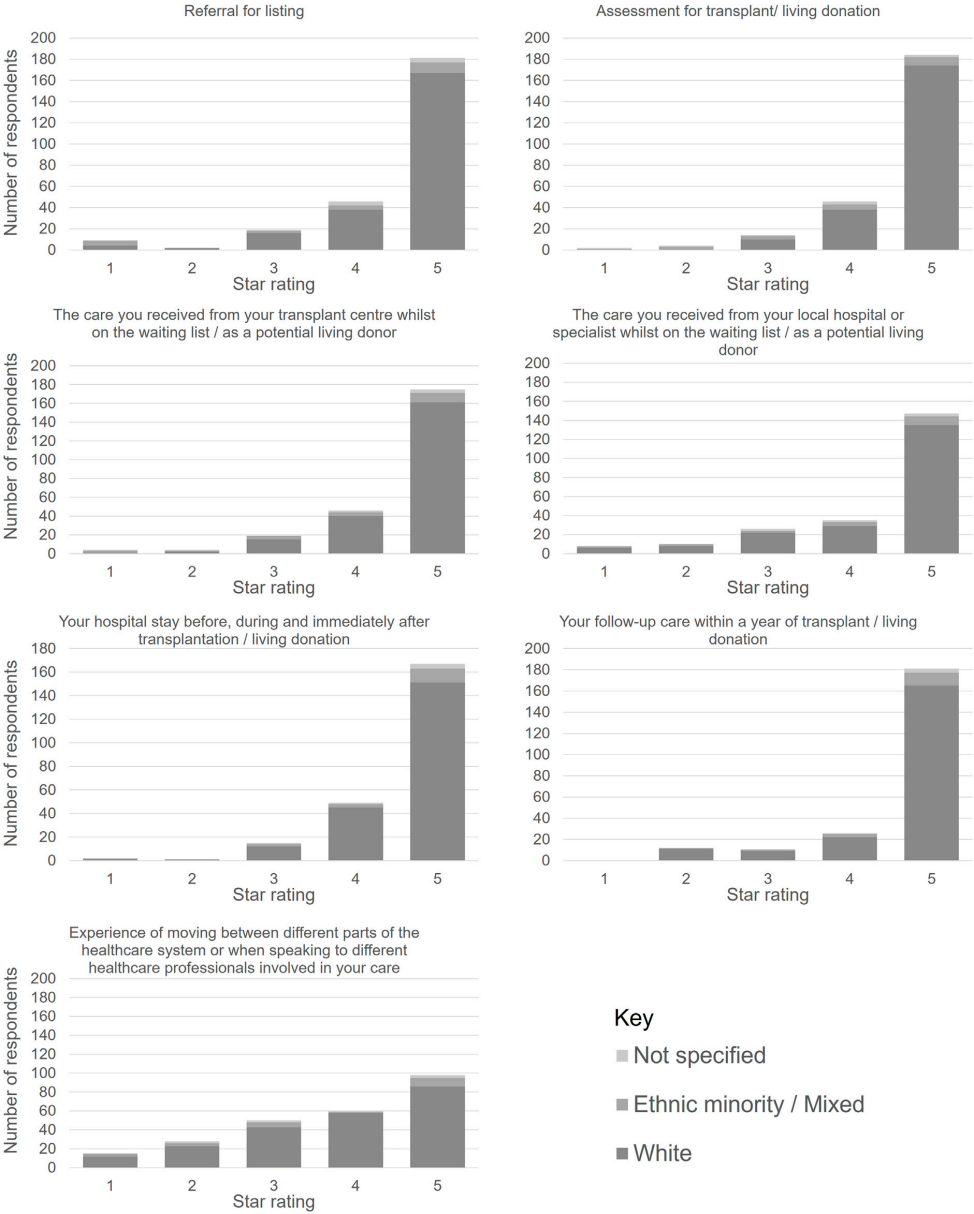
Responses to the online patient survey with satisfaction rates for care received along the transplant care pathway.

A summary of the responses received to the survey is provided in Figure 3. Overall, patients were very satisfied with the levels of care received along the care pathway. The only exception was their experience of moving between different service providers as they progressed along the care pathway.

The OUG held online patient focus groups in 2021, Chaired by OUG patient representatives. Delegates were invited via patient representative groups. To support open, honest feedback, participants were anonymous, and feedback was not attributable to any specific patient. There were 27 participants across 4 focus groups as noted in Table 3.

**TABLE 3:** OUG focus group participants.

Focus group	Organ type	Participants
Focus Group 1	Kidney	1 Asian; 5 Black; 2 White delegates
		1 parent of paediatric patient with special needs​
		1 representative of adult special needs patient
		2 male and 6 female delegates
Focus Group 2	Lung	5 White delegates
		1 male and 4 female delegates
		Pre- and post-transplant
		1 patient who had been a child at the time of listing
Focus Group 2	Kidney	6 Black delegates
		2 male and 4 female delegates
		Pre- and post-transplant
		2 delegates on the waiting list
Focus Group 4	Liver	8 White delegates
		4 male and 4 female delegates
		Pre- and post-transplant
		Experience of transplant during COVID

The OUG was keen to hear from “less heard voices”—particularly female, Black, and Asian patients. The Group experienced challenges in finding people willing to discuss their transplant experience. Patient representative groups were approached to identify people to participate, and patients and family members/carers were self-selected. It is therefore possible that the experiences and views raised may not be representative of the wider patient population, as most patients who participated had experienced specific challenges that they wished to raise and were confident in highlighting their experiences. Their self-selective nature of participation meant that some groups were strongly skewed towards particular conditions, which may not be representative of the wider patient pathway.

### Feedback From Those Involved in Delivering the Transplant Service

**FIGURE 4:**
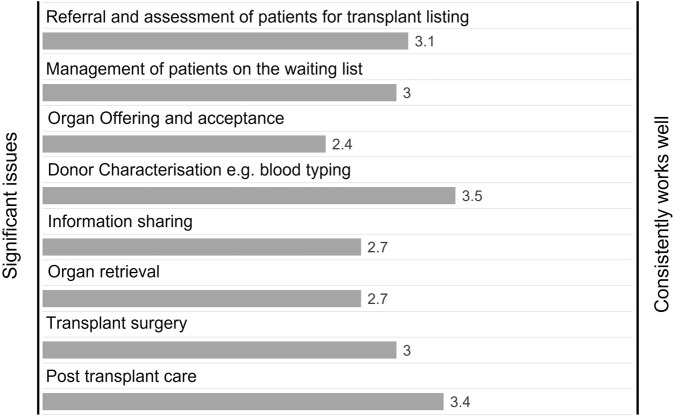
Workshop delegate responses to the question “Rate the current performance of aspects of the transplant system” (*n* = 71).

**FIGURE 5:**
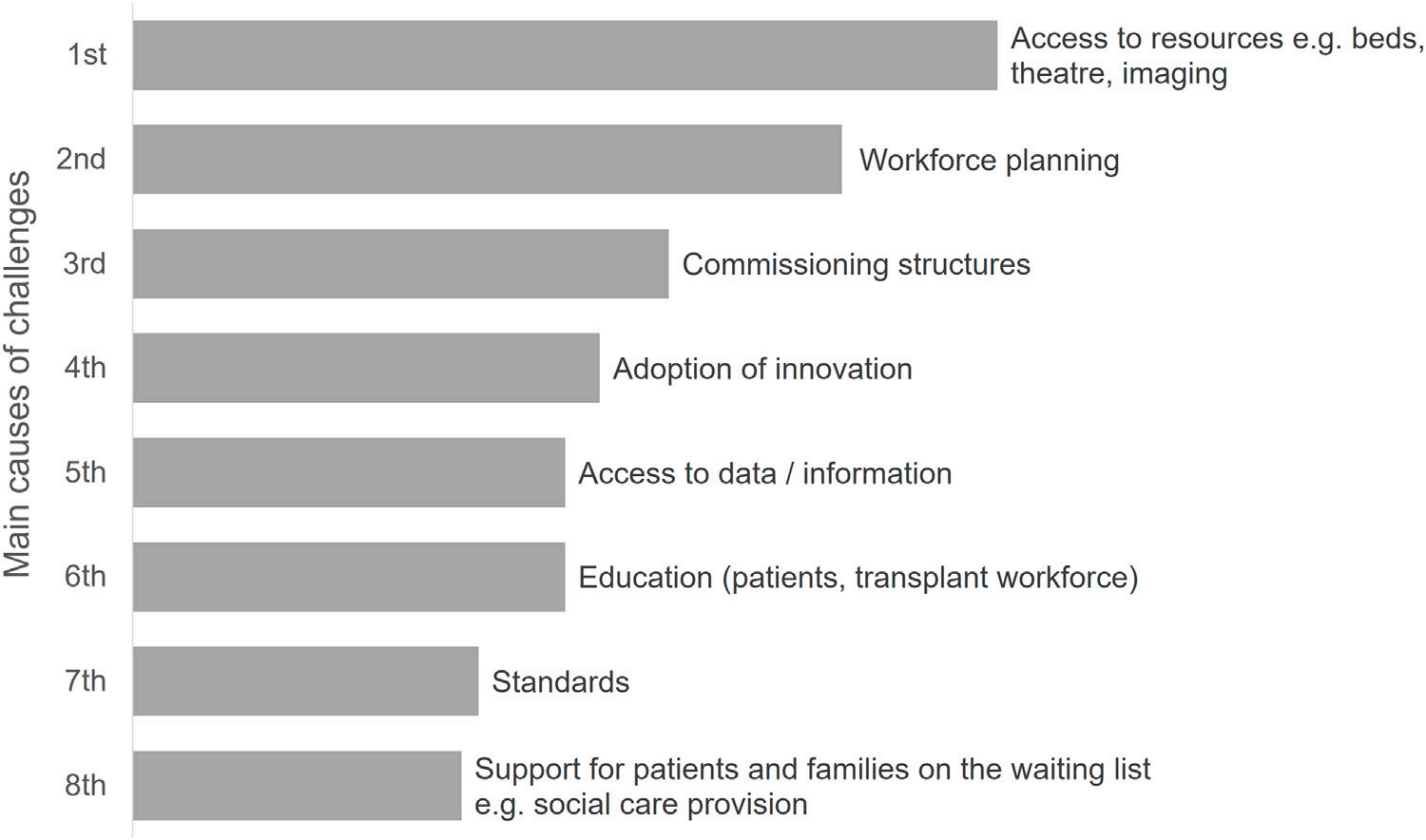
Workshop delegate responses to the question “What do you consider are the main causes of challenges in organ transplantation?” (*n* = 71).

The OUG held an online workshop in October 2021, to provide stakeholders with the opportunity to advise on the key challenges and opportunities in transplant services. Delegates were invited to use the online voting mechanism “Mentimeter” [8], with 71 delegates participating in the voting. Delegates were asked to rate the performance of aspects of the transplant system using a sliding scale, where ratings closer to the left indicate significant issues/difficulties with a specific service that needed to be addressed. Ratings closer to the right would mean that the service consistently worked well. Delegates identified “organ offering and acceptance”, “information sharing” and “organ retrieval” as having the most significant issues. There were no areas that delegates advised were consistently working well (see Figure 4). Respondents identified resourcing, workforce, patient support and technology as the key issues and challenges (see Figure 5 for more information).

Contact was made with donation and transplant experts in 7 countries (Austria, Australia, Canada, France, Netherlands, Spain, and the USA). It was agreed that the issues described in the UK were often present in these other countries—further collaborative work is planned [9]. The following key similarities were noted:

• Maximising utilisation potential.
• Risk appetite and centre variation—not possible to eliminate, but should seek to reduce the amplitude.
• Utilisation rates driven by local enthusiasts.
• Few instances of any national level oversight of the whole care pathway.
• Workforce burnout and recruitment/retention issues especially post-pandemic.

Supplementary Table S2 in the Supplementary Material provides a summary of the responses including lessons learned and successful initiatives.

An online meeting was held with UK national clinical leads for transplant services to seek views on the challenges and opportunities. These focussed on four key areas:

i. Trust (Individual Hospital) involvement—there was a need for hospital Boards to take ownership of the issues regarding transplantation and do more to support both patients and clinical teams.
ii. Addressing risk aversion—there was a need to do more to support those who take reasonable risks and address logistical barriers to those who are willing to accept higher-risk organs.
iii. Workforce—transplant teams have to work unsociable hours with little reward. There was an increasing trend for surgeons to leave the UK to work in other countries.
iv. Resources—Intensive care capacity was raised as a particular issue to be addressed.

More detailed feedback is provided in Supplementary Table S3 of the Supplementary Material.

**FIGURE 6:**
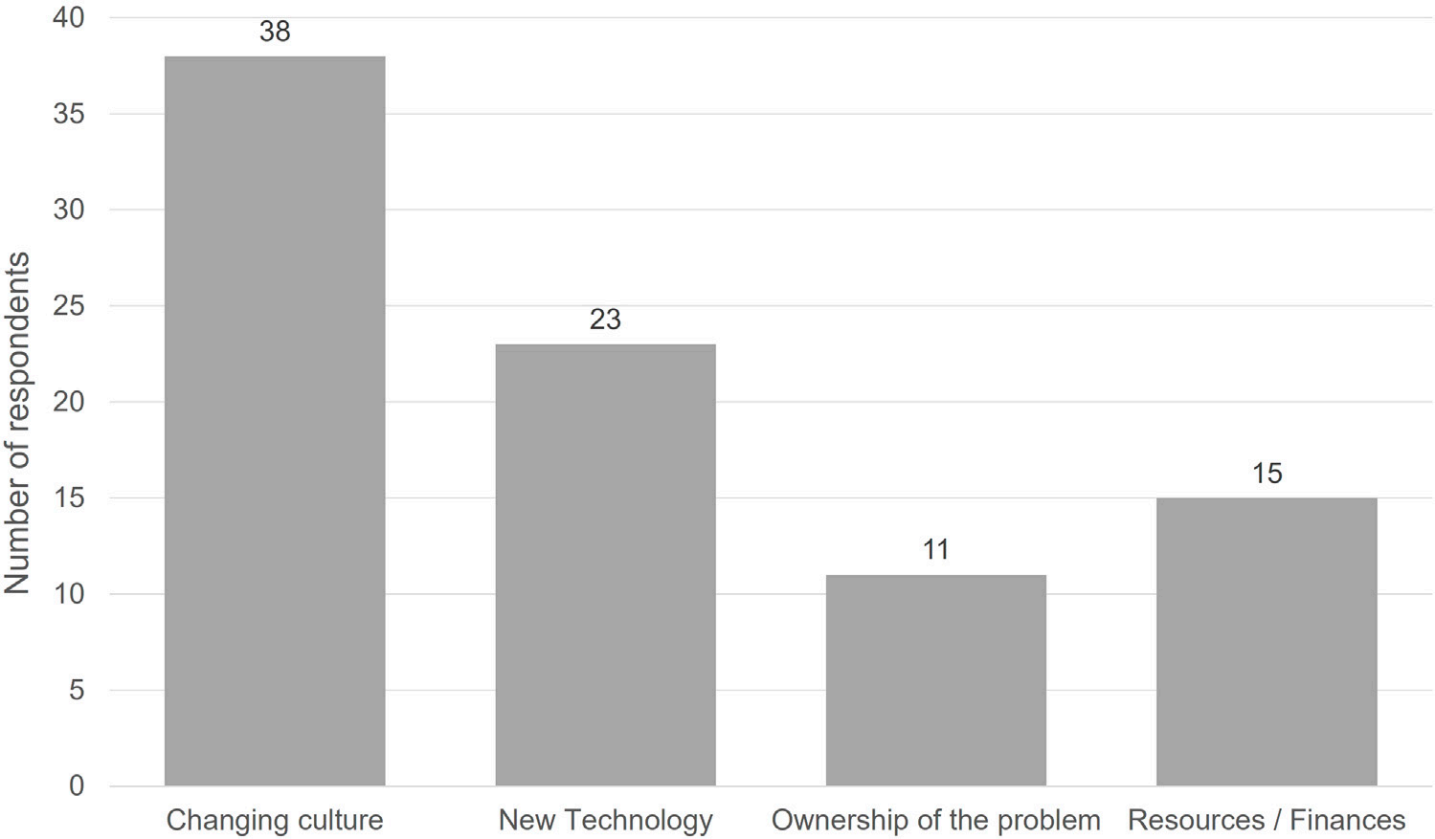
National Organ Utilisation Conference delegate responses to the question “What change would have the most positive impact on organ utilisation?”

Views from clinical teams were also sought at the UK National Organ Utilisation conference in May 2022. Delegates included representatives from across the transplant service. Delegates gave a clear steer that changing the culture would have the greatest impact on organ utilisation (see Figure 6).

The majority of patients raised the importance of psychological and social care support and where this was lacking, the negative impact on experience for patients and their families, and patient outcomes. Patients expressed frustration regarding poor communication. This included the timeliness of communication and the lack of effective communication along the care pathway. For example, a lack of timely, effective sharing of notes between different providers. Patients noted that there was inconsistency in advice received—particularly relating to medication and diet—between different providers and regions. This caused concern and anxiety. Female patients also noted a lack of available advice regarding issues such as sexual and reproductive health.

Those delivering the transplant service noted concerns in the workforce, particularly relating to staff fatigue, difficulties in recruitment and the high rate of staff attrition. They raised frustration at the lack of ability to quickly adopt proven innovation and machine perfusion technologies as standard practice, noting that this was limiting the numbers of organs that could be utilised. There was significant variation in access to theatres, beds, and key staff, which limited a hospital’s opportunity to accept offered organs. The length of the donation, offering, and allocation process was also a cause of frustration and limited the ability to forward plan and secure local resources for the transplant procedure.

**FIGURE 7:**
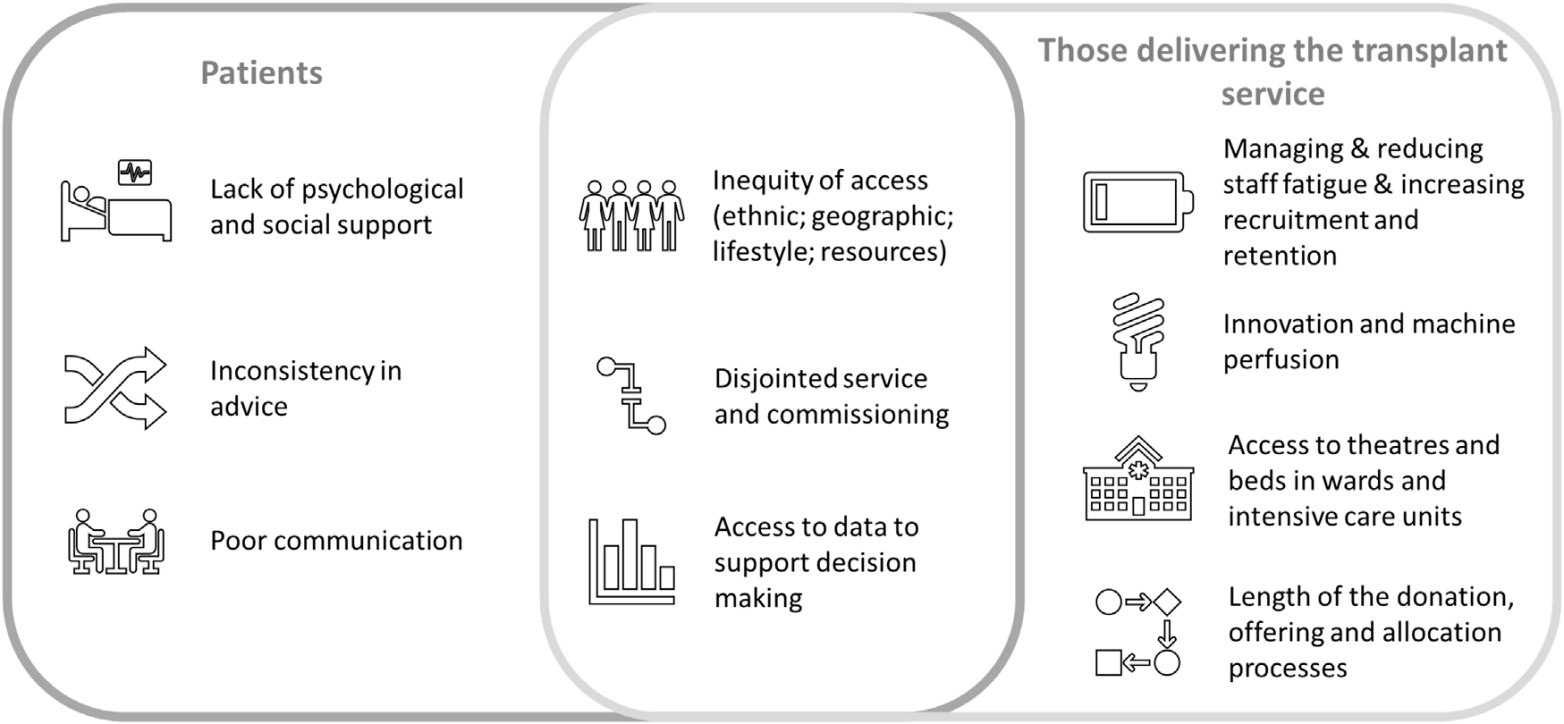
Summary of issues in transplantation, raised by patients and those delivering the transplant service.

Both groups expressed concern regarding inequity of access to transplantation services across a range of factors, including ethnic, geographic, lifestyle, and resources. There were concerns regarding the disjointed service along the care pathway, which they believed could be partly attributable to having to move between different commissioners and providers. Finally, they advised that there was a lack of timely access to data to support their decision-making regarding organ acceptance. For patients, this included the need to ensure that data was provided in an easily understandable format, tailored to meet their needs. A summary of the issues in transplantation raised by patients and those delivering the service is provided in Figure 7.

### Statistical Evidence

**FIGURE 8:**
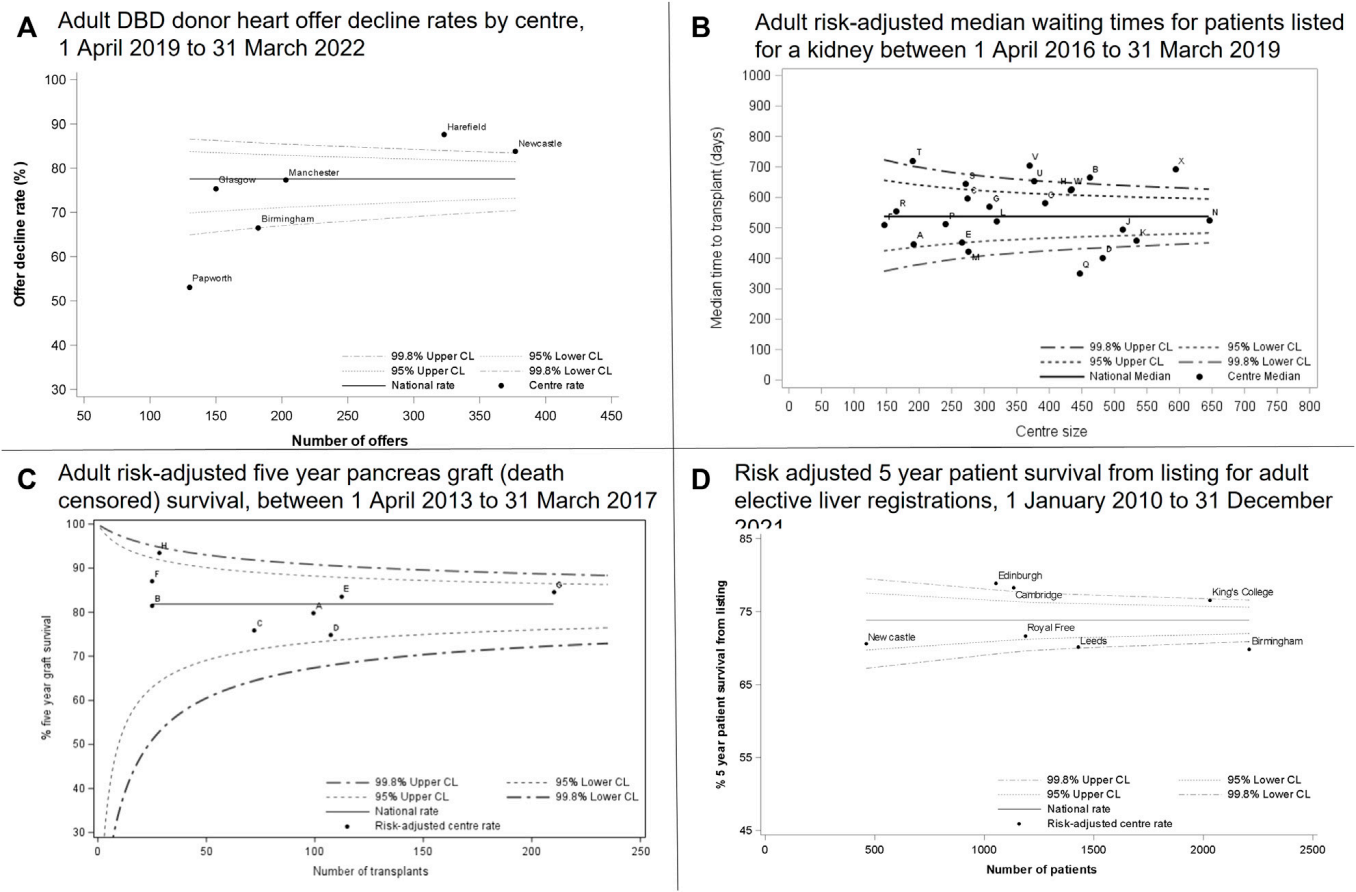
Centre variation across offer decline rates, risk-adjusted median waiting times, graft survival and patient survival.

There is unwarranted variation in access to transplantation, organ acceptance and post-transplant survival leading to inequities in care and treatment for patients. Figure 8 highlights the differences between centres for various stages in the transplant pathway for all organs. Each chart shows a funnel plot for the different outcomes displayed. The average rate for the UK is shown as the horizontal thick black line with the dotted lines representing the upper and lower confidence limits. Each centre is represented by a dot. Where a centre falls above or below the dotted lines, this indicates that the centre has a statistically higher or lower than average rate compared to the UK rate. Chart A shows DBD donor heart offer decline rates ranging from 53% to 88% across centres. This shows a difference in appetite for accepting donor offers between centres. Chart B shows risk-adjusted median waiting times for patients listed for a kidney transplant. Risk-adjusted median waiting times range from 1 to 2 years across centres even after adjusting for patient demographics. Variation in acceptance rates can lead to unwarranted variation in waiting times. Chart C shows risk-adjusted five-year pancreas death censored-graft survival ranging from 75% to 93%. Although no centre has significantly poorer outcomes compared with the national rate, the variation can still impact the length of time a patients graft functions. Chart D shows 5-year risk-adjusted patient survival from listing for adult elective liver patients and ranges from 70% to 79% across the centres. It is important not only to look at post-transplant survival, but also survival from listing as this accounts for deaths on the waiting list as well as deaths post-transplant. Similar graphs are available for other organs in the NHSBT annual report [10] and demonstrate a similar pattern.

### OUG Vision and Recommendations

The OUG final report includes a vision for transplant services, which focusses on the following issues [11]:

• To ensure a donated organ is transplanted into the intended recipient as rapidly as possible, through delivering a service that is:
  • Supporting and empowering patients (improved data that enables patients to understand their options and reflects the diversity of those on the transplant waiting list; giving patients a louder voice in shaping the services that they rely on).
  • Equitable (regardless of geography, socio-economic status, health literacy, culture or ethnicity).
  • Reducing unwarranted variations in practice (clearer expectations about roles and responsibilities; infrastructure enables adherence to best practice).
  • Driving cost savings to the NHS (increasing the number of transplants; maximising the efficient use of available resources).
  • Honouring the gift from donors (no opportunity missed for safely transplanting an organ into the intended recipient).
  • Supporting and empowering transplant teams (data, guidance and training provided in a timely, accessible manner).
  • Sustainable (resources; workforce).
  • Embedding innovation (supporting new techniques, technologies and evidence-based best practices).
  • Placing the UK as a world leader (organ transplant rates; forefront of research).

The published report [9] provides 12 recommendations spread across the following themes:

1. Placing the patients at the heart of the service.
2. An operational infrastructure that maximises transplant potential
3. Creating a sustainable workforce that is fit for the future.
4. Data provision that informs decisions and drives improvements.
5. Driving and supporting innovation.
6. Delivering improvements through new strategic and commissioning frameworks.

The recommendations provide imperatives for activity, with accompanying support actions to inform implementation (for details see Supplementary Table S4 in the Supplementary Material).

### Implementation

**FIGURE 9:**
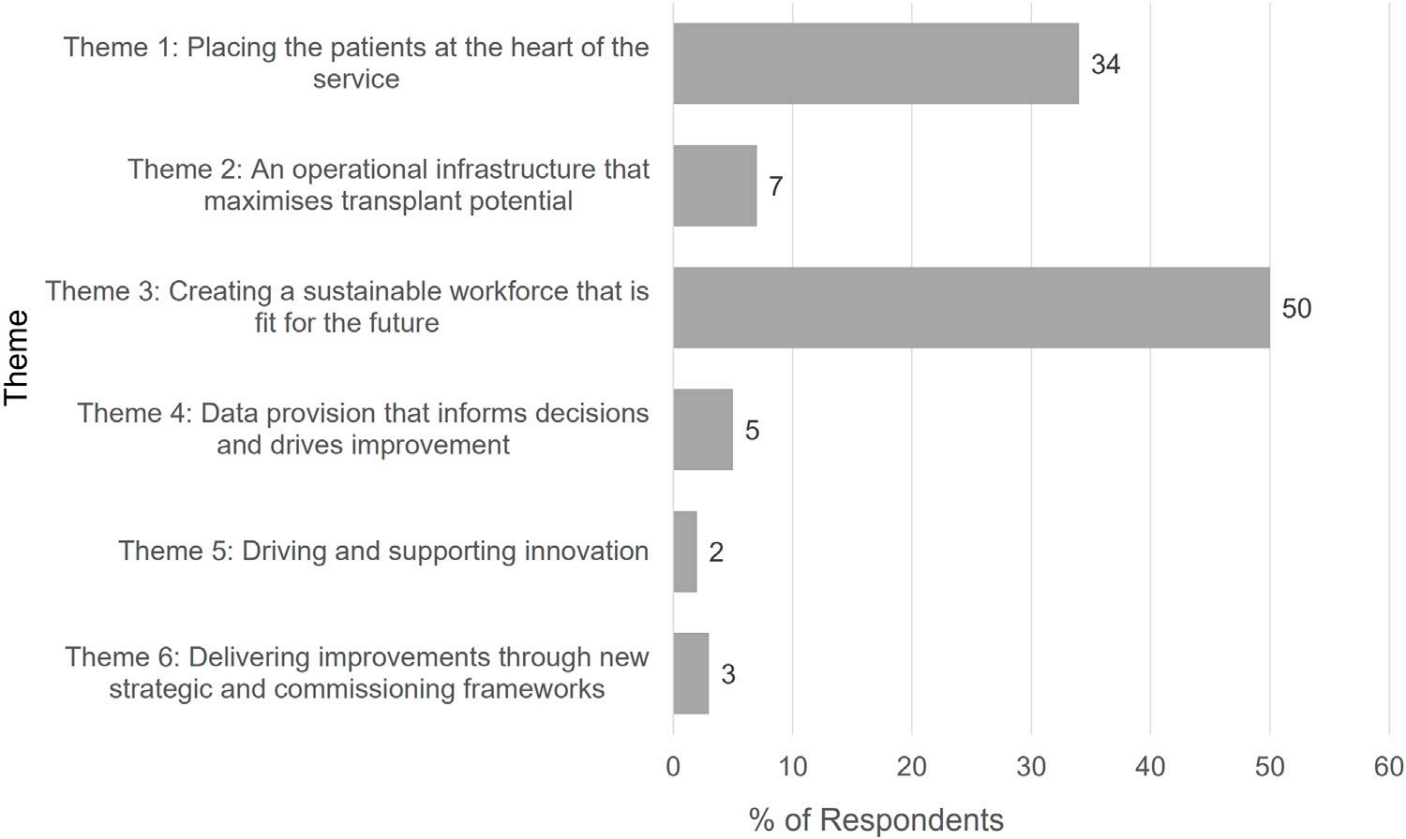
Feedback from British Transplantation Society Conference Delegates to the Question “Which theme (from the OUG report) do you believe should be given the highest priority?”

Feedback has been sought regarding the priorities for implementation. At the OUG seminar of the 2023 British Transplantation Society Annual Congress, delegates were asked to use an online survey tool to identify which of the report’s themes should be given the highest priority. Delegates were only able to choose one theme. The responses received are provided in Figure 9, demonstrating that the need to create a sustainable workforce was considered the highest priority (50% of respondents), closely followed by placing patients at the heart of the service (34% of respondents).

**FIGURE 10:**
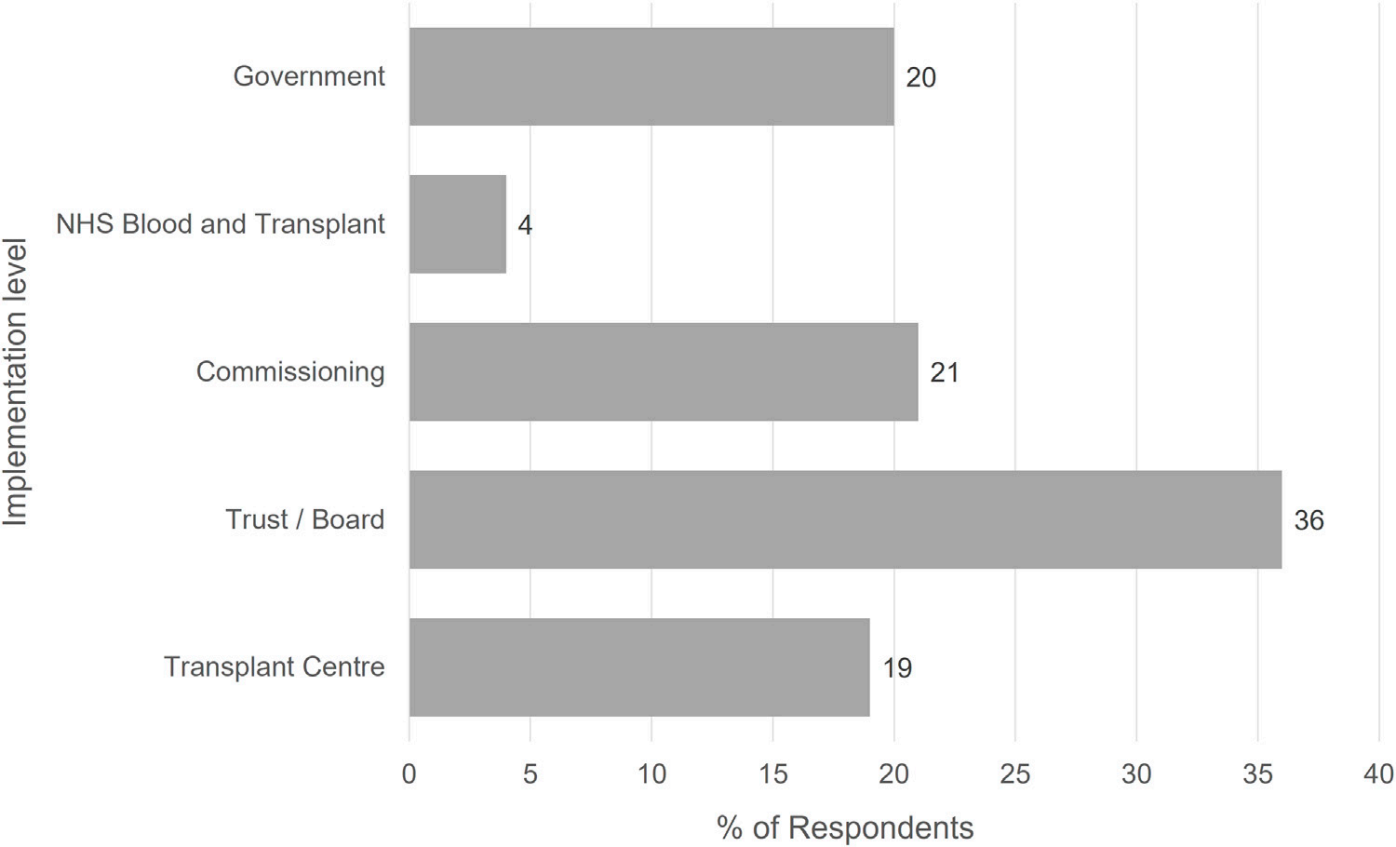
Feedback from British Transplantation Society Conference Delegates to the Question “At what level are the biggest challenges to implementing the OUG recommendations?”

Delegates were asked which of the main groups responsible for implementation of the OUG recommendations would have the biggest challenges. The responses are provided in Figure 10, demonstrating that Government, Commissioning, and Transplant centres were all identified as being equally challenging, but the highest level of challenge would be with local NHS Trust engagement and implementation.

### Initial Impact of the OUG

The OUG work has already started to have an impact and deliver a number of benefits to transplant services.

The establishment of the OUG demonstrated a renewed interest in organ utilisation from the Government, with an aim to maximise the potential benefit of introducing Opt Out legislation and save more lives through the gift of organ donation. The publication of the report was accompanied by a Written Ministerial Statement from Minister Neil O’Brien, Parliamentary Under Secretary of State (Minister for Primary Care and Public Health). This included commitments to implementing the recommendations in full and delivering improvements to the transplant service [12].

**FIGURE 11:**
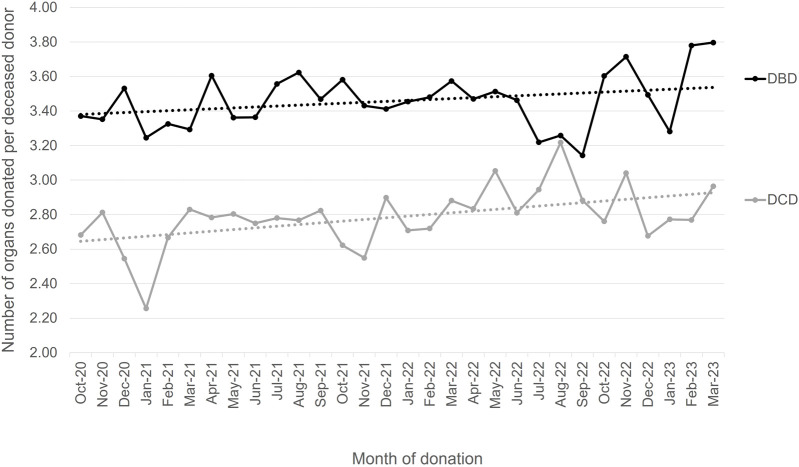
Number of organs per donor by month and donor type with trendlines, 1 October 2020–31 March 2023.

Figure 11 shows the number of organs donated per donor and donor type for each month since the start of the Clinical Leads for Utilisation (CLU) schemes through to the OUG report publication and beyond. The superimposed linear trendlines show an increase by month for both DBD and DCD donors in the numbers of organs retrieved for the purpose of transplantation, with DCD increasing at a higher rate. Although not directly attributable, the CLU schemes along with directed interest in organ utilisation would have positively contributed to the increases seen.

## Discussion

The OUG report sets out a new strategy and direction for transplant services. It builds on a range of existing national and local initiatives, bringing them together to provide revised impetus, strategic direction, and national oversight. It also identifies a range of areas for new focus within transplantation, such as improvements in personalised care for patients.

The recommendations place the patient at the heart of the service and seek to honour the gift of donation through ensuring that organs are transplanted in a safe and equitable manner. The report aims to improve the experience of both service providers and users and improve utilisation rates.

The level of engagement with the OUG work—national and international—demonstrates the acknowledgement of transplant teams, providers, and patients, in the need for change and a will to work together to deliver improvements. The fact that the OUG was a Government initiative, with the report published by Ministers, will help to ensure national focus on implementation. However, the high levels of stakeholder engagement through the report development stages need to be maintained—indeed is even more important—for the implementation stage.

The limitations to the project included the difficulty in surveying all patient attitudes. Rather than surveys with a set threshold response rate, the evidence was sought via all major relevant patient groups, often encouraging anonymous reporting if that facilitated engagement. Specific focus groups, aided by leaders of Ethnic Minority groups, helped in listening to “less heard voices” but coverage may not have been comprehensive. The scope of this project, partly by design to examine transplant processes, and partly the logistics of a report that was achievable, were limited and did not seek to include issues at referral nor in donation. That is not to say that these are not important; far from it. But the thrust of the report and evidence gathering is around the processes of transplantation.

The recommendations and supporting actions within the OUG report are complex and require action by multiple organisations, at national, regional, and local levels. The OUG remit was to deliver recommendations that would take up to 5 years to deliver. It will take time to map the multiple co-dependencies both across the 6 themes of the report and also with other work underway nationally and locally. This will support the identification of priorities for action.

It is acknowledged by all involved in this project that publication of a report will change little without recommendations being carried forward to support this work. Therefore, the English Department of Health and Social Care Ministers have established the Implementation Steering group for Organ Utilisation (ISOU), to bring together those with a role in implementation, agree priorities and timescales and then monitor and support implementation. Other countries in the UK have observers on this group and have indicated that they wish to carry out implementation in a similar manner. The group has senior policy and clinical Co-Chairs and membership includes providers, commissioners, patient and lay representation, as well as subject matter experts. The first ISOU meeting was held in April 2023—less than 2 months from the publication of the report—demonstrating the Government’s commitment to implement the recommendations as quickly as possible.

There is work underway within ISOU to develop an implementation plan, with supporting Key Performance Indicators, to monitor progress and impact of the implementation approach. If successful, the following benefits will be realised:

• Increase in utilisation rates.
• Improved equity of access.
• Decrease in current rates of higher quality declines or lack of resources declines.
• Improved patient experience.
• Improved patient engagement.

## Figures and Tables

**Table:** 

Members		
Role	Title	Name
Chair	Prof Sir	Stephen Powis
Deputy Chair	Prof	John Forsythe
Trust Chief Executive	Mr	Julian Hartley
Trust Chief Executive	Mr	Stephen Posey
Critical Care Clinical Director	Dr	Gus Vincent
Organ utilisation – abdominal	Mr	Chris Callaghan
Organ utilisation – cardiothoracic	Mr	Steven Tsui
Recipient transplant co-ordinator	Ms	Moira Perrin
Director of operations	Dr	Maurice Hakkak
British Transplantation Society	Mr	Krish Menon
Living donation	Ms	Lisa Burnapp
Lay representative	Mr	Shamik Ghosh
Patient representative - kidney	Ms	Hilaria Asumu
Patient representative - CT	Ms	Jessica Jones
Non-transplant centre	Dr	David Makanjuola
Departmental representative	Mr	Michael Gallagher
Departmental representative	Ms	Maria Nyberg
Lead Secretariat	Ms	Ms Claire Williment
Secretariat - Data and Statistics	Ms	Lisa Mumford
Secretariat support	Mr	Danielle Fothergill
Secretariat support	Ms	Andrea Pereira
Secretariat support	Ms	Cathy Hassell

Observers		
Role	Title	Name
Devolved Government representative	Ms	Caroline Lewis
Devolved Government representative	Ms	Joan Hardy
Devolved Government representative	Ms	Sharon Grant
England Specialised Commissioning	Ms	Fiona Marley
England Specialised Commissioning	Ms	Sarah Watson
Commissioning	Ms	Anushka Govias-Smith
Commissioning	Mr	Stuart Davies
Commissioning	Ms	Teresa Magirr
Commissioning	Ms	Karen Quinn
Stakeholder Forum Chair	Ms	Fiona Loud
Stakeholder Forum Chair	Prof	Deirdre Kelly

## Data Availability

The original contributions presented in the study are included in the article/Supplementary Material, further inquiries can be directed to the corresponding author.
