# Supplementary material for: Excellence in Organ Utilisation—A Quantitative and Qualitative Evidence Base for a New Approach in the UK
Source: Transpl Int. 2023 Sep 4;36:11641. doi: 10.3389/ti.2023.11641 (PMC10505655; doi:10.3389/ti.2023.11641)
Supplement: Supplementary file 1 [file Table1.docx]

**Table 1: Summary of delegate workshop regarding key challenges and issues in transplantation**

| **Theme** | **Points raised** |
| --- | --- |
| Resources | - Access to theatres and beds - exacerbated by Covid backlogs - Workforce shortages - Surgeon burnout - Access and equity within organisation - Attracting nurses into the discipline due to the hours - Resource constraints - The time it takes to arrange deceased donation - Lack of available organs - Overnight work - Difficulty in getting the organs transported - Trust prioritising access to transplants - Capacity to provide transplantation - Managing resources in view of other priorities in the Trust - Inequitable access - Delays in waiting for the retrieval team to arrive - ‘Business as usual’ (BAU) at donor and recipient centres - Potential benefits could be seen in aligning the organ donation and transplant paths to allow for greater utilisation and addressing common issues, such as access to theatres or Trust/ Board-level engagement with issues and progress. |
| Technology | - Funding for novel technology - Electronic data capture - Sharing information - Organ assessment - Infrastructure |
| Patient | - Referrals to charity to facilitate peer support - Lack of donors/ donation from paediatric deceased donors under 5 years - Variation of acceptance of organs - Optimal donor care - Emotional headspace after transplant - Addressing inequality - There are national and local barriers. Organ donation is not aligned with transplantation needs and there is a disconnect between the two. I am mainly referring to logistics. - Satisfying the need for donor organs: the waiting lists are artificially limited and the real need is enormous - Engaging with ethnically diverse communities - Matching sensitised patients - Getting patients worked up and active on the transplant list - Cultural barriers - Health inequalities |
| Supporting patients | - Need to empower patients to take an active role in their own care. - Benefits could be achieved if information is available at an early stage of the journey (it is suspected that patients often know transplantation is inevitable) - Increased access to correct, concise, accessible information may help patients take a more active role in their own care. - Access to services to support patients pre- and post-transplant needs to be considered, particularly social care and psychological support - Potential issue with mistrust around transplantation among ethnic minorities. There was a suggestion that more could be done to engage with ethnic minorities. |
| Workforce | - Work-life balance attitude seems to be changing and long hours associated with transplantation are unattractive - May need to engage employees within every part of the pathways so impact can be realised e.g., surgeons see patients in aftercare (emphasis that every part of the pathways is equally important) - It is important to be able to rapidly mobilise staff - Issues with understaffing |
| Other | - Funding - Fighting for priority amongst other health priorities - Overcomplicated commissioning pathway - Expanding transplant workforce and infrastructure - Need to learn from Covid and mutual aid between transplant units to help address any local resource issues. |
