# Supplementary figures and images for: Excellence in Organ Utilisation—A Quantitative and Qualitative Evidence Base for a New Approach in the UK
Source: Transpl Int. 2023 Sep 4;36:11641. doi: 10.3389/ti.2023.11641 (PMC10505655; doi:10.3389/ti.2023.11641)

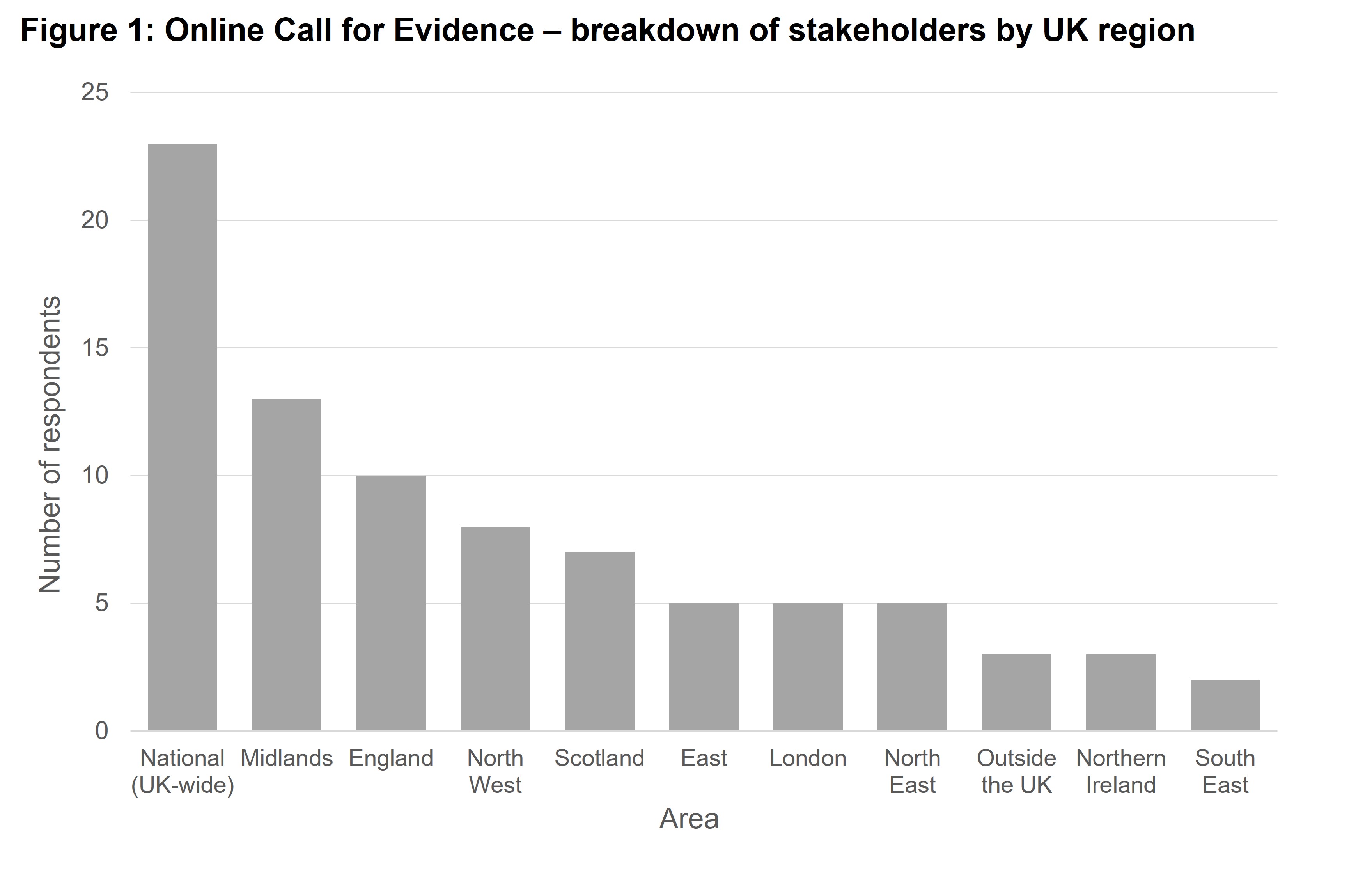

Supplement: Supplementary file 2 [file Image1.jpeg]

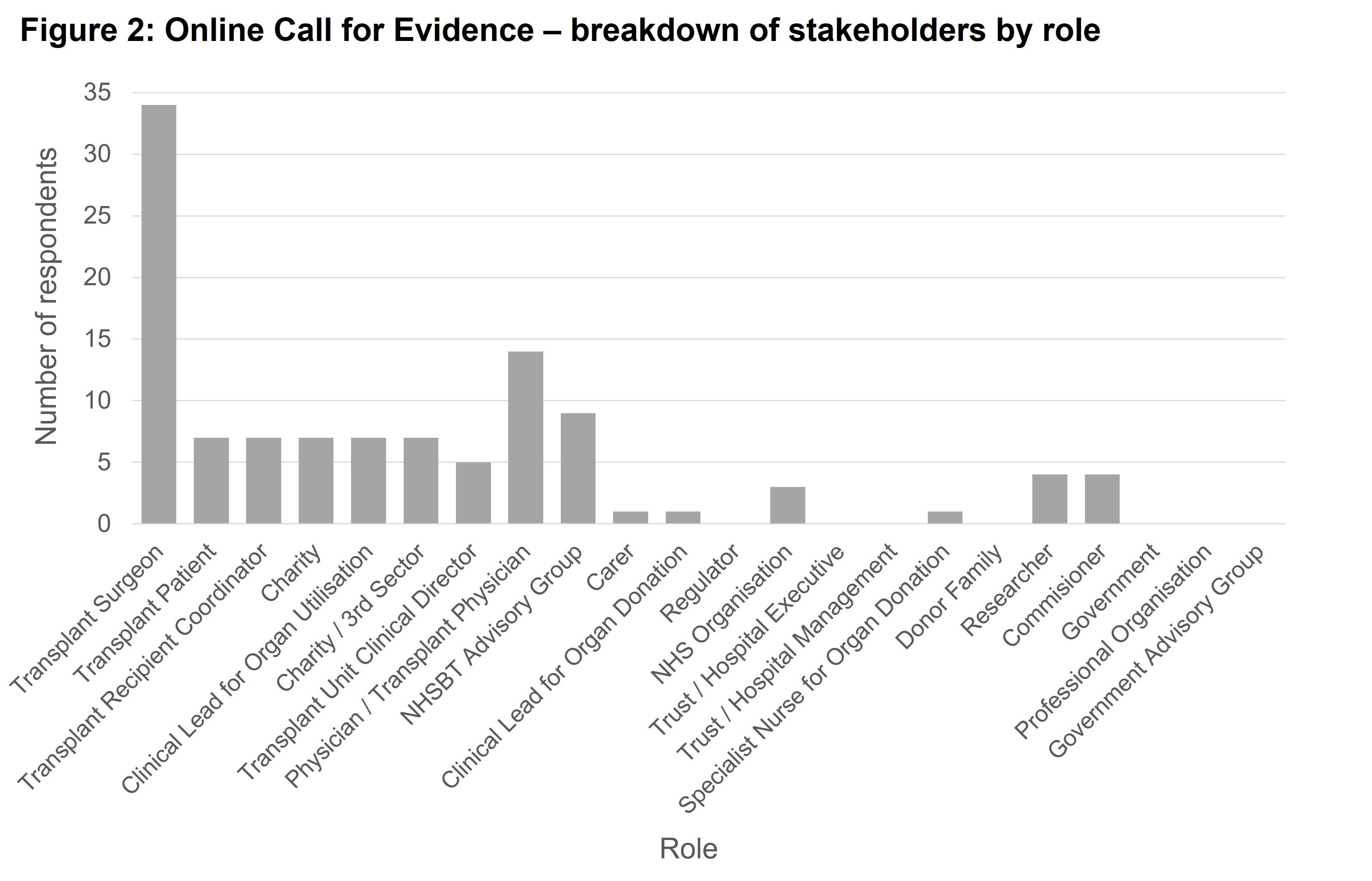

Supplement: Supplementary file 3 [file Image2.jpeg]
