# Supplementary material for: Excellence in Organ Utilisation—A Quantitative and Qualitative Evidence Base for a New Approach in the UK
Source: Transpl Int. 2023 Sep 4;36:11641. doi: 10.3389/ti.2023.11641 (PMC10505655; doi:10.3389/ti.2023.11641)
Supplement: Supplementary file 4 [file Table2.docx]

**Table 2: Summary of responses from international meetings**

| **Theme** | **Feedback** |
| --- | --- |
| Similarities between countries | - Maximising utilisation potential - Risk appetite and centre variation – not possible to eliminate, but should seek to reduce the curve - Utilisation rates driven by local enthusiasts - Few instances of any national level oversight of the whole care pathway - Workforce burnout and recruitment/ retention issues |
| Lessons learned from adverse experiences | - 70% acceptance rates in one region for hearts, but no quality control on outcomes - Reporting measures that focus on chastising poor outcomes increases risk averse behaviour - A large number of centres means increase in transplant, competition etc, but also means that some centres have very low transplant rates due to small waiting lists |
| Lessons learned from successful initiatives | - Having agreements, preferably formal contracts, in place regarding resource requirements minimised organ declines - Always have 2^nd^ opinion regarding an organ decline - Set measures that incentivise - Focus on monitoring adherence to best practice, rather than only criticising best practice - Targets/ KPIs for waiting lists. Waiting lists managed and mutual aid given to gain balance across units. Managed by centres, not nationally. Protocols in place to trigger national involvement when necessary - Have metrics that are patient-focussed – supports patient involvement and incentivises Units - External scrutiny for severely underperforming Units, with Trust management involved - Benchmarking and outcome measures are key to driving improvements - Monitor activity and risk averse behaviour and provide annual training programmes to address any common issues and share experience and best practice - Reference centres for complex cases or concerns regarding risk/ safety issues, with specialist on call 24/7 to provide advice designated by the Health Department as a specialist service - If a centre accepts a high-risk organ, then they don’t go to the bottom of the list for the next organ - Support those who take risks. Don’t disincentivise those who accept higher risk donors. Challenge those who don’t accept standard criteria donors |
| Supporting innovation | - Developing predictive analytics to address risk aversion and prevent inappropriate offers - Use Global Positioning System (GPS) tracking of organs in transit, to support resource planning and inform future improvements - Standardise biopsy pictures - Establishing a new matching system to help those with longer wait and highly sensitised patients, with built in simulation to explore likely outcome. - All dialysis patients are required to be considered for transplant within 12 months. - Undertaking a mapping exercise to look at donation and transplantation across the country, to identify problems and solutions - 70% of Donors after Circulatory Death (DCD) donors have Normothermic Regional Perfusion (NRP). Looking to establish DCD Hearts and Thoraco-Abdominal NRP - Provides a mobile extracorporeal membrane oxygenation (ECMO) team for DCD procedures - Focussing on shared decision making with patients - Using advanced tissue typing to ensure organ goes to the right patient, with machine perfusion to ensure it is in the best possible state |
