# Supplementary material for: Excellence in Organ Utilisation—A Quantitative and Qualitative Evidence Base for a New Approach in the UK
Source: Transpl Int. 2023 Sep 4;36:11641. doi: 10.3389/ti.2023.11641 (PMC10505655; doi:10.3389/ti.2023.11641)
Supplement: Supplementary file 5 [file Table3.docx]

**Table 3: Summary of responses from an online meeting with national leads for transplant services**

| **Theme** | **Feedback** |
| --- | --- |
| NHS Trust Board involvement/ ownership | - Need KPIs/ service spec to drive change and focus attention - Remuneration for transplantation based on actual activity, with performance monitoring - Support patients to engage with data and ‘Vote with their feet’ |
| Addressing risk aversion | - ‘It’s not risk aversion. It’s the pain that comes after a bad outcome from a risk taken. Impacts on a personal level, particularly when you have to speak to the family’ - Need more support for those taking risks - Need to remove logistical barriers before addressing risk aversion – needs resources, education and support - Support patients to understand their own risk appetite through benchmarking - CUSUM triggers are a disincentive – need reviewing - Need psychological advice/ review regarding how to address risk averse behaviour - Conflict between having local waiting lists and national offering – doesn’t account for differences in centre size and a disincentive for those with a small waiting list. - Have a named clinician in every centre to undertake benchmarking for each clinical decision - Improve data about outcomes of organs declined for one centre and accepted by another |
| Workforce | - Often have to work in middle of the night with hostile, unwilling people (e.g., theatre teams) - Need to find better ways to reward staff. - Need to engage and motivate those teams not so closely involved in transplant (e.g., theatre teams) and make them feel part of the transplant community. |
| Resources | - Smaller units should band together to share and manage resources - Need to engage with intensivists about transplant issues and enable the process to be streamlined - ICU is supportive but struggles to manage due to the uncertainty of transplantation timings. |
