# Supplementary material for: Excellence in Organ Utilisation—A Quantitative and Qualitative Evidence Base for a New Approach in the UK
Source: Transpl Int. 2023 Sep 4;36:11641. doi: 10.3389/ti.2023.11641 (PMC10505655; doi:10.3389/ti.2023.11641)
Supplement: Supplementary file 6 [file Table4.docx]

**Table 4: Recommendations and supporting action within the Organ Utilisation Group Report**

| **Recommendation** | **Supporting actions** |
| --- | --- |
| **Theme 1: Placing the patient at the heart of the service** | |
| 1. Patients who are being considered for transplantation, referral or listing must be supported and have equal access to services irrespective of personal circumstances, including ethnic, geographical, socio-economic status or sex | 1.1 Communication with patients must be provided in a timely manner and in a format that is easily accessible, understandable and appropriate to the patient’s needs. Each transplant centre must provide local relevant data for patients and supports them in understanding and engaging with the information provided. |
|  | 1.2 Patients must be supported to understand the care options that are available, both in different forms of transplant (for example living or deceased donation) and alternatives to transplant. |
|  | 1.3 Patients must be able to access information about their local centre performance in comparison with other accessible centres |
| 1. Transplant services must be run with reference to patient feedback, including frequent opportunities to listen and act on views from less heard voices. | 2.1 Patient preference must be taken into consideration early in the referral process when determining where a transplant may occur, acknowledging that the location may change – potentially at short notice – to ensure that the patient receives a transplant in timely fashion. |
|  | 2.2 Any service development must be co-produced with users of the service, including patients, their carers and clinicians. |
|  | 2.3 Evaluation of live donor’s/ live and deceased donor family’s/ recipient’s experience and outcomes must be undertaken at all stages of the care pathway. |
|  | 2.4 Patients must regularly meet with clinical teams, to provide feedback on the service received. This is particularly relevant for ‘less heard voices’. |
|  | 2.5 Patient Reported Outcome Measures (PROMs) and Patient Reported Experience Measures (PREMs) must be subject to similar levels of focus and scrutiny as clinical outcomes. Measures must be co-produced with patients and co-publicised with patient representative groups. |
| Theme 2: **An operational infrastructure that maximises transplant potential:** Standardised pathways | |
| 1. Standardised patient pathways must be developed and made available for each organ type, with well-defined timescales for each stage of the pathway. Data available for each stage of the pathway informs monitoring against best practice. Clinical Leads for Utilisation support the review of the data, to identify and drive local improvement initiatives. | 3.1 Decline meetings in transplant units must be established as a mandatory requirement, with a nationally agreed profile and template. |
|  | 3.2 Service delivery standards must be produced to provide clarity on the roles and timelines for each of the steps in the care pathway relating to patient assessment for transplantation and placement on the waiting list. |
|  | 3.3 Standards must be developed to support the removal of non-clinical reasons, such as the lack of an available theatre, as a valid cause for organ offer decline and make them an extraordinary event. Patients must be able to find out if an organ has been declined on their behalf due to a lack of resources, should they wish to do so. |
|  | 3.4 These standards must be inspected and monitored by commissioning reviews carried out jointly by NHSE and NHSBT, with requisite and appropriate data made available from relevant parties, including NHSBT and the NHS Trust |
|  | 3.5 All referring centres must record a decision regarding referral for transplant assessment within one month of presentation of a patient with end-stage organ failure. |
|  | 3.6 Every unit must have a Clinical Lead for Utilisation, responsible for data oversight and monitoring within their unit, working with clinical and management colleagues to deliver improvements. |
| **Theme 2: An operational infrastructure that maximises transplant potential:** Sustainability of the service | |
| 1. Transplant units must build on the lessons learned during the COVID-19 pandemic and increase further the collaborative effort across units. | 4.1 All units must regularly meet and discuss organ acceptance and decline activity to share learning, best practice and data as follows: |
|  | 4.2 Refined and improved outcome data from NHSBT on organs declined must be developed and disseminated, to provide better data-driven prediction on the possible performance of a particular donor organ. |
|  | 4.3 The above decline detail must form part of the regular commissioning review. |
| 1. NHSE must undertake a comprehensive review of cardiothoracic services to ensure that services in place are sufficiently sustainable and resilient and are able to provide the best possible outcome for patients. | 5.1 NHSE Specialised Commissioning must work closely with NHSBT and the relevant patient and professional organisations to ensure that the review has the necessary insight and expertise. |
|  | 5.2 International benchmarking and patient outcome data, held by NHSBT must be included in the evidence base for the review. |
| **Theme 3: Creating a sustainable workforce that is fit for the future** | |
| 1. A National Transplant Workforce Template must be developed to provide definitions of the skill mix for an effective, safe and resilient transplant workforce that is fit for current and future demands. | 6.1 There must be workforce planning toolkits for all forms of transplantation to support workforce planning and reduce inequities across the service. The number of personnel at each centre would be defined by local demographics, such as waiting list size, catchment areas and so on. However, the expertise required are consistent throughout and Annex 3 provides the minimum skills. Algorithms could be developed to support the planning activity. |
|  | 6.2 Psychological and social care support must be available for patients both around the time of transplant and in follow up. The annual review for patients on the waiting list must include a review of psychological and social care support requirements, tailored to meet the needs of the patient. For referral, transplant and follow-up services, consideration is given regarding support for patients when treatment is far away from their home. |
| **Theme 4: Data provisions that informs decisions and drives improvements** | |
| 1. The provision of data must be transformed, using digital approaches to provide access to complete, accurate and standardised data and information to everyone who needs it at critical decision points throughout the donation to transplantation pathway. | 7.1 The information and data sources required at each stage of the transplant care pathway for different users must be identified and provided. |
|  | 7.2 Assessment must be made of the feasibility of creating a user-centred ‘portal’ that integrates all data and information, with priority being given to the user-group and/ or stage of the pathway that will drive the biggest improvements to organ utilisation. |
|  | 7.3 The availability and use of tools to support patients and clinicians in their discussions about transplant options and potential impact on patient outcomes (for example waiting times) must be improved. |
|  | 7.4 Data terminology, collection and secure transfer processes must be standardised across the UK, to ensure completeness, accuracy and accessibility of data, including access to patient data for multiple transplant centres. Building on existing knowledge and infrastructure: |
|  | 7.5 The relevant data in donation and transplant pathways must be digitised to enable efficient and accessible flow of data from point of recording to point of access: |
| **Theme 5: Driving and supporting innovation** | |
| 1. National multi-organ centres for organ assessment and repair prior to transplantation must be established to provide the optimum practical steps to bring new techniques into everyday clinical therapy as rapidly as possible, to maximise the number and quality of organs available for transplant and support logistics at transplant units. | 8.1 The centres must eventually cover all organ types, with initial focus on lung and liver transplantation. |
| 1. A national oversight system must be established that makes the best use of the UK's world leading innovation in assessment, perfusion and preservation of donated organs. | - 1. There must be a system to provide oversight and alignment, which is particularly relevant for:   - perfusion that starts and/ or occurs in-situ, such as Donation after Circulatory Death (DCD) Hearts and Normothermic regional perfusion (NRP)   - innovation and novel therapies where there is a need for national consideration for the clinical safety and ethics, such as xenotransplantation, genomics and lab-based techniques for altering the DNA of an organism |
|  | 9.2 The oversight system must be used to address inequity of access that results from the variations in clinical involvement and resource availability. |
|  | 9.3The system must move units up the learning curve as rapidly as possible, to maximise the potential for improving organ transplantation. |
| **Theme 6: Delivering improvements through new strategic and commissioning frameworks:** Strategic direction and oversight | |
| 1. All NHS trusts with a transplant programme must have a transplant utilisation strategy to maximise organ utilisation. | 10.1 A Board member must be responsible for production and regular (at least annual) Board review of this strategy. The review includes patient feedback and input. |
|  | 10.2 NHSBT must regularly provide summary data, in a standardised template, to enable the Trust board to review progress against their own strategy. |
|  | 10.3 The strategy must be jointly inspected at least annually by NHSE and NHSBT. |
| 1. National measurable outcomes must be defined and agreed in order to prioritise, monitor and evaluate the success of key strategies, tools and processes. | 11.1 There must be a definition of “optimal” organ utilisation. |
|  | 11.2 There must be an evaluation of donors/ donor families/ recipients experience and outcomes at all stages of the care pathway including living donation transplant procedures. |
|  | 11.3 Factors of health inequality must be monitored to ensure equity of access. |
|  | 11.4 Techniques must be established to enable donors, donor families, recipients and clinicians to understand and use measurable outcomes. |
| **Theme 6: Delivering improvements through new strategic and commissioning frameworks:** Commissioning | |
| 1. Robust commissioning frameworks must be in place, with well-defined roles and responsibilities of the various agencies involved in organ transplantation, particularly focusing on the relationship between NHSBT and Commissioners. Memorandums of Understanding (MoUs) across the agencies must be created to formalise the process for the joint commissioning of transplant services. | 12.1 There must be well-defined service specifications, containing national standards to drive service improvement and support performance management, recognising the whole patient pathway. The specifications must underpin the commissioning activity. The metrics must enable the evaluation of outcomes, innovation and future service development. |
|  | 12.2 MoUs must be established to provide clarity on the roles and responsibilities of providers at each stage of the care pathway and indicate how different providers will collaborate to provide an effective service, as well as at which points patients will move from one provider to another for care. |
|  | 12.3 A financial framework must be in place, which encompasses a standardised approach to costing the patient pathway and service provider reimbursement, optimising transplantation. Periodic modelling of future demand supports resource planning. |
